# Maternal high-fat diet and risk of cardiovascular disease in offspring: a review of mechanisms, controversies, and interventions

**DOI:** 10.3389/ebm.2026.11199

**Published:** 2026-09-01

**Authors:** Heyu Meng, Jianjun Ruan, Qing Wang, Fanbo Meng

**Affiliations:** 1 Department of Endocrinology, China-Japan Union Hospital of Jilin University, Jilin University, Changchun, China; 2 Department of Cardiology, China-Japan Union Hospital of Jilin University, Jilin University, Changchun, China

**Keywords:** coronary artery disease, developmental origins of health and disease (DOHaD), high-fat diet, maternal exposure, offspring

## Abstract

A high-fat diet (HFD) is a well-established risk factor for coronary artery disease (CAD). Accumulating evidence indicates that maternal nutritional status before and during pregnancy critically influences long-term cardiovascular outcomes in offspring. This review systematically synthesizes current mechanistic, epidemiological, and clinical evidence regarding the impact of HFD on CAD while also examining its effects on broader cardiovascular phenotypes that are integral to CAD pathogenesis, such as endothelial dysfunction, cardiac remodeling, and metabolic derangements. A particular focus is placed on the potential effects of maternal pre-pregnancy HFD exposure on offspring CAD risk in adulthood. Existing studies suggest that HFD induces coronary injury primarily through metabolic dysregulation, endothelial dysfunction, and epigenetic reprogramming. Notably, maternal exposure to HFD during the perinatal period may predispose offspring to coronary artery disease via these mechanisms. Conversely, appropriate maternal nutritional interventions during pregnancy have shown promise in mitigating these adverse effects, although their long-term efficacy and underlying molecular mechanisms remain to be fully elucidated. Future research should prioritize large-scale prospective cohort studies with extended follow-up to determine the optimal timing, mechanisms, and efficacy of dietary interventions. Such evidence would provide a scientific foundation for developing preventive and therapeutic strategies aimed at reducing cardiovascular disease risk from the earliest developmental stages.

## Impact statement

Although maternal diet during pregnancy is known to affect child health, the long-term impact of maternal pre-pregnancy high-fat diet on offspring coronary artery disease in adulthood has not been systematically synthesized. This review fills this critical gap by focusing explicitly on the preconception period, offering important implications for early-life prevention of coronary heart disease. This review integrates multi-level evidence from animal studies, epidemiology, and clinical research to construct a comprehensive mechanistic framework involving metabolic dysregulation, endothelial dysfunction, and epigenetic reprogramming. Importantly, it does not evade existing controversies and conflicting findings, thereby clarifying priority research questions for future investigations. This review identifies that coronary microvascular injury is an early pathological event preceding myocardial dysfunction in high-fat diet-induced cardiac damage. Furthermore, it systematically compares the differential effects of maternal pre-pregnancy versus pregnancy dietary patterns and exercise interventions on offspring cardiovascular outcomes, providing detailed mechanistic and outcome-based associations. By identifying coronary microvascular injury as an early and reversible pathological event that precedes heart muscle dysfunction, this work shifts the field's focus toward earlier diagnostic targets and preventive windows. It also highlights that maternal diet before conception—not just during pregnancy—plays a critical programming role in offspring coronary health. Furthermore, by clearly mapping existing evidence gaps and controversies, this review provides a roadmap for future large-scale cohort studies and mechanism-driven interventions. Ultimately, these insights lay the groundwork for developing evidence-based, life-course strategies to reduce coronary artery disease risk from the very earliest stages of development.

## Introduction

Coronary artery disease (CAD) remains a leading cause of mortality and morbidity globally. Its pathogenesis is closely associated with various lifestyle factors, particularly dietary composition [1]. A high-fat diet (HFD), which is characteristic of the Western dietary pattern, has been widely associated with dyslipidemia, endothelial dysfunction, and atherosclerotic plaque formation—key pathological processes that increase the risk of CHD [2, 3]. Current research has increasingly focused on the long-term impact of maternal nutrition before and during pregnancy on offspring cardiovascular outcomes, guided by the “Developmental Origins of Health and Disease” concept [4, 5]. Maternal exposure to HFD has been shown to induce metabolic and epigenetic alterations in the developing fetus, thereby predisposing offspring to lipid metabolism disorders, insulin resistance, and early vascular injury. Comprehensive investigation of the mechanistic link between HFD—specifically maternal HFD exposure during the perinatal period—and offspring coronary health is of significant clinical and public health relevance. Such investigations hold potential for improving early prevention strategies and risk stratification of CHD.

## Pathological mechanism and long-term risk analysis of coronary artery damage caused by a high-fat diet

### Physiological basis and pathological changes of coronary health

Maintenance of coronary health is contingent upon the structural and functional integrity of the coronary vasculature. Coronary arteries, which supply oxygen and nutrients to the myocardium, rely on finely regulated vasomotor activity that ensures adequate blood perfusion to meet the metabolic demands of cardiac tissue. Under physiological conditions, the coronary microvascular plays a crucial role in regulating myocardial blood flow and metabolic homeostasis, maintaining a balance between oxygen delivery and cardiac metabolic demand. However, pathological changes that compromise coronary structure or function, such as atherosclerosis, lead to vascular stenosis, obstructed blood flow, and consequent myocardial dysfunction [1].

**FIGURE 1:**
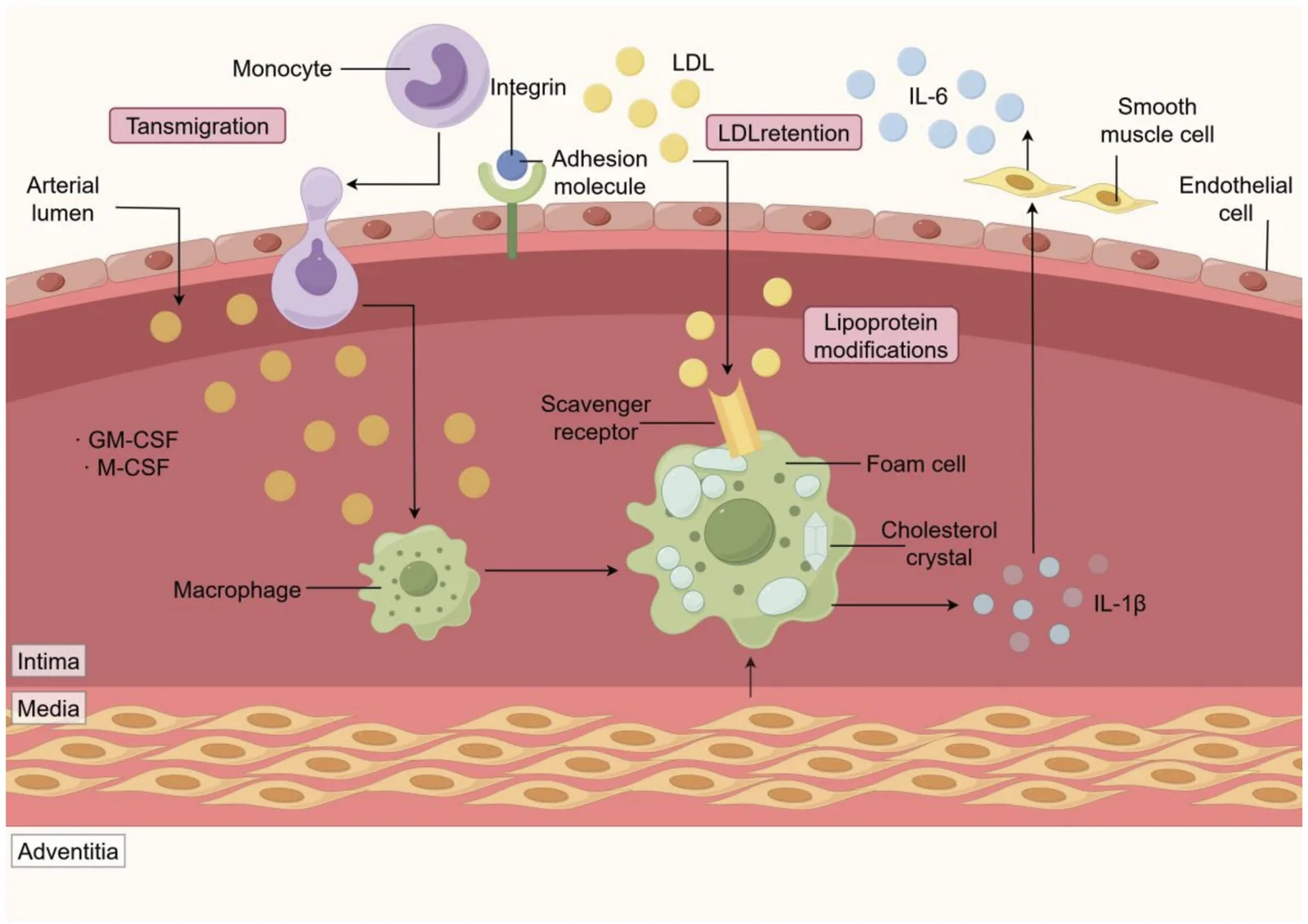
Cellular and molecular mechanisms of early atherosclerotic plaque formation. The schematic illustrates the initiation of atherosclerosis driven by subendothelial LDL retention and modification. Monocytes tether to endothelial adhesion molecules and transmigrate into the intima, differentiating into macrophages under the influence of GM-CSF and M-CSF. Macrophages engulf modified lipoproteins through scavenger receptors and are transformed into cholesterol crystal-laden foam cells. The subsequent secretion of pro-inflammatory cytokines by foam cells, notably IL-1β, amplifies local intimal inflammation, alters medial smooth muscle cell activity, and drives the release of IL-6.

In the arterial lumen, circulating low-density lipoprotein (LDL) crosses the endothelial cell barrier and enters the intima, where it undergoes LDL retention, followed by a series of lipoprotein modifications. At the same time, monocytes in the blood recognize and bind to adhesion molecules on endothelial cells through their surface integrins, completing transendothelial migration into the inner membrane microenvironment. In the endometrium, monocytes differentiate into macrophages under the influence of local factors such as GM-CSF and M-CSF. Subsequently, the macrophages ingest a large amount of modified lipoproteins through the scavenger receptor on its surface, leading to lipid overload in cells and the formation of cholesterol crystals; this eventually causes them to transform into foam cells. These accumulated foam cells further secrete proinflammatory cytokines, as represented by IL-1β. This persistent inflammatory microenvironment not only affects smooth muscle cells, but it also promotes the release of inflammatory mediators, such as IL-6, into the bloodstream, thereby cascading and amplifying the pathological inflammatory response throughout the entire vascular wall (see Figure 1).

In disease states, progressive structural and functional changes within the coronary vasculature drive the development of ischemic heart disease. The accumulation of atherosclerotic plaques promotes vascular stenosis, which restricts coronary perfusion and can lead to myocardial ischemia and tissue injury. The severity of coronary stenosis has been shown to correlate strongly with the occurrence of myocardial ischemia and adverse cardiac events; when this stenosis exceeds a critical threshold, it severely restricts myocardial blood flow, resulting in angina pectoris or myocardial infarction [6]. Cardiac remodeling, particularly myocardial hypertrophy, represents another hallmark of cardiac coronary pathology and can be classified as either physiological or pathological. Physiological myocardial hypertrophy occurs under normal conditions such as growth, pregnancy, or athletic training, and is associated with preserved or enhanced cardiac function. In contrast, pathological hypertrophy arises in response to chronic stressors, such as hypertension, myocardial infarction, or neurohormonal activation, and is characterized by maladaptive features, including interstitial fibrosis, capillary rarefaction, increased pro-inflammatory cytokine production, and endothelial cell dysfunction. These changes ultimately culminate in cardiac remodeling and heart failure (HF) [7]. Collectively, these physiological and pathological insights underscore the intricate balance required for maintaining coronary health and emphasize the importance of early interventions aimed at preserving endothelial function, preventing atherosclerotic progression, and mitigating maladaptive cardiac remodeling.

### Mechanisms of high-fat diet effects on the cardiovascular system

#### Effects of a high-fat diet on the cardiovascular system through hemodynamics

Animal experimental studies further confirm the detrimental effects of HFD on the cardiovascular system and coronary function, with pathophysiological effects involving multiple mechanisms, including metabolic, molecular, and functional alterations. Maternal HFD exposure during pregnancy significantly affects the cardiovascular structure and function in offspring: for example, in C57BL/6 female mice, maternal HFD during pregnancy (pHFD) resulted in male offspring that, at 8 weeks of age, exhibited baseline cardiovascular function comparable to controls alongside attenuated responses to isoproterenol-induced stress, with cardiac output, stroke volume, and left ventricular fractional shortening reduced by 20%–45%; in contrast, female offspring showed reduced baseline cardiovascular parameters but maintained a normal stress response [2]. The mechanism of isoproterenol-induced cardiovascular stress can be summarized into the following four aspects: 1) Cardiac overstimulation: the strong activation of cardiac β receptors results in marked increases in heart rate and strong contractility, leading to a sharp increase in myocardial oxygen consumption, oxygen supply–demand imbalance, and subsequent myocardial ischemic injury; 2) Calcium overload and oxidative damage: calcium ion overload and the generation of a large amount of reactive oxygen species directly damage myocardial cells, thereby triggering cell apoptosis and inflammatory reactions; 3) Activation of the renin-angiotensin-aldosterone system (RAAS): indirect activation of the RAAS causes water and sodium retention, vasoconstriction, and myocardial fibrosis, thereby increasing cardiac workload; 4) Vasodilation and blood flow disorders: dilating peripheral blood vessels leads to a significant decrease in blood pressure (especially diastolic blood pressure), reflexively exacerbating tachycardia and placing the heart in a high-output, low-resistance state, which can easily induce HF or shock.

**FIGURE 2:**
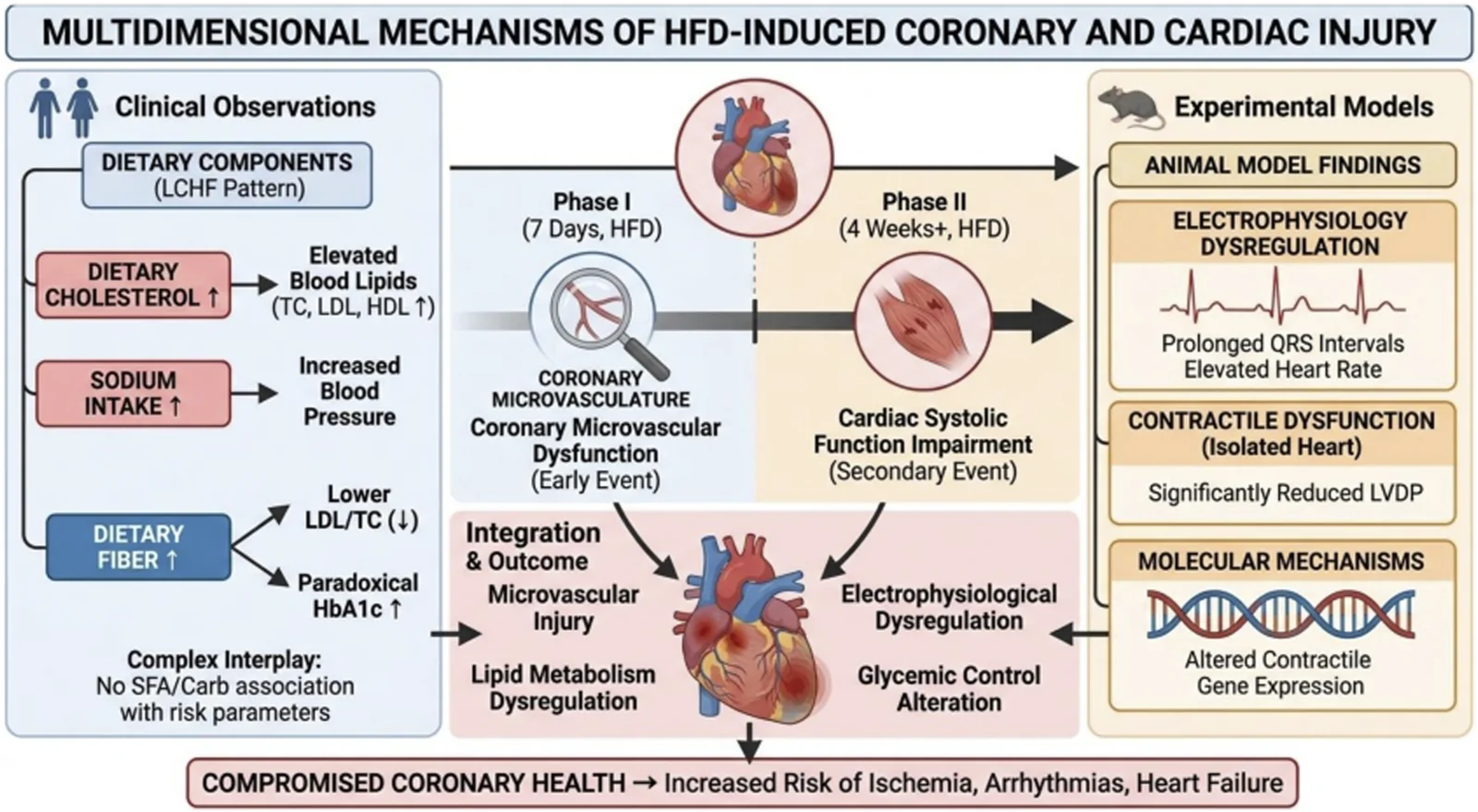
Animal experimental studies confirming the impact of HFD on coronary artery function. The intake of HFD leads to significant cardiac dysfunction, manifested as prolonged QRS interval, increased heart rate, and significantly reduced LVDP. Coronary microvascular injury is an early pathological event before myocardial dysfunction in HFD-induced cardiac injury.

Similarly, in a hereditary hypertriglyceridemic rat model (pregnancy high-fat model), an 8-week high-fat, high-fructose diet (HFFD) resulted in electrocardiographic abnormalities (including prolonged QRS complex and tachycardia), along with significantly suppressed baseline cardiac contractility and reduced left ventricular developed pressure (LVDP) [3]. In terms of animal models directly exposed to HFD, hereditary hypertriglyceridemic rats fed an HFFD exhibited significant cardiac dysfunction, characterized by prolonged QRS intervals, an elevated heart rate, and significantly reduced LVDP in isolated heart perfusion experiments, accompanied by pronounced alterations in the expression profiles of genes related to cardiac contraction [2]; in murine models, short-term HFD containing 60% fat and 1% cholesterol induced severe coronary microvascular dysfunction within 7 days, whereas impairment in cardiac systolic function became evident only after 4 weeks, suggesting that coronary microvascular injury was an early pathological event preceding myocardial dysfunction in HFD-induced cardiac injury [8] (Figure 2). Collectively, these preclinical findings indicate that HFD may compromise coronary health through pathways involving cardiac electrophysiological disturbances, impaired myocardial contractility, and microvascular injury [9–12]; a summary of these findings is presented in Table 1.

**TABLE 1:** Clinical and experimental observations on the effects of high-fat diet on coronary health.

Study type/subject	Maternal diet/intervention	Core findings	Conclusion/mechanistic insight	Citation
Human observational study	LCHF diet	Dietary cholesterol increased TC, LDL, and HDL levels; dietary sodium intake elevated BP; dietary fiber decreased LDL and TC but increased HbA1c	The effects of LCHF diets on lipid metabolism are complex and require individualized monitoring; sodium restriction is critical for BP regulation	[13]
Animal study (rats)	HFFD	ECG abnormalities (prolonged QRS interval and increased heart rate); significantly decreased LVDP in isolated hearts	HFFD directly impairs cardiac electrophysiology and contractility, potentially mediated through altered through gene expression and metabolic stress	[13]
1. Hereditary hypertriacylglycerolemic rats	HFFD for 8 weeks	ECG abnormalities, including prolonged QRS interval and heart rate, as well as significantly reduced LVDP in isolated hearts	HFFD directly impairs cardiac electrophysiology and contractile function through lipid accumulation and metabolic stress	[3]
2. Metabolic syndrome rat model	High-fat/high-sucrose diet + puerarin supplementation	Puerarin administration improved glycemic control, lipid profile, and blood pressure; reduced cardiac and arterial remodeling, neural damage, and inflammation	Puerarin mitigates HFD-induced cardiometabolic dysfunction through anti-inflammatory and antioxidative pathways	[14]
3. Male Wistar rats	HFD + L-arginine supplementation	L-arginine reduced cardiac lipid and glucose accumulation	Mechanism involves the suppression of leptin levels and enhancement of PIK3 signaling activity	[15]
Animal study (mice)	Short-term HFD (60% fat +1% chol.)	Coronary microvascular dysfunction was detected within 7 days, preceding cardiac systolic changes observed after 4 weeks	Coronary microvascular dysfunction represents an early event in HFD-induced cardiac injury and may serve as an early predictive indicator	[16]
1. C57BL/6 female mice and their offspring	Prenatal HFD (pHFD)	Male offspring exhibited attenuated cardiac stress response (20%–45% reduction in cardiac output); female offspring showed reduced baseline cardiovascular parameters	Maternal HFD induces sex-specific developmental programming that impairs cardiovascular function in offspring	[2]

#### Effects of a high-fat diet on the cardiovascular system through metabolic disturbances and alterations in gene expression (excluding hemodynamic effects)

Beyond hemodynamic dysfunction, HFD significantly disrupts cardiac metabolism and alters gene expression. In rats with high-fat/high-sucrose diet-induced metabolic syndrome, puerarin administration significantly improved glycemic and lipid profiles, reduced blood pressure, and attenuated cardiac and vascular remodeling, neural injury, and systemic inflammation [14]. Similarly, L-arginine supplementation in male Wistar rats fed an HFD reduced cardiac lipid and glucose accumulation by decreasing circulating leptin levels and promoting phosphoinositide 3-kinase (PIK3) activity [15]. These findings demonstrate that HFD induces cardiovascular dysfunction through multiple mechanisms that impair cardiac stress response, disrupt metabolism, and alter gene expression related to cardiac metabolism and vascular homeostasis.

**FIGURE 3:**
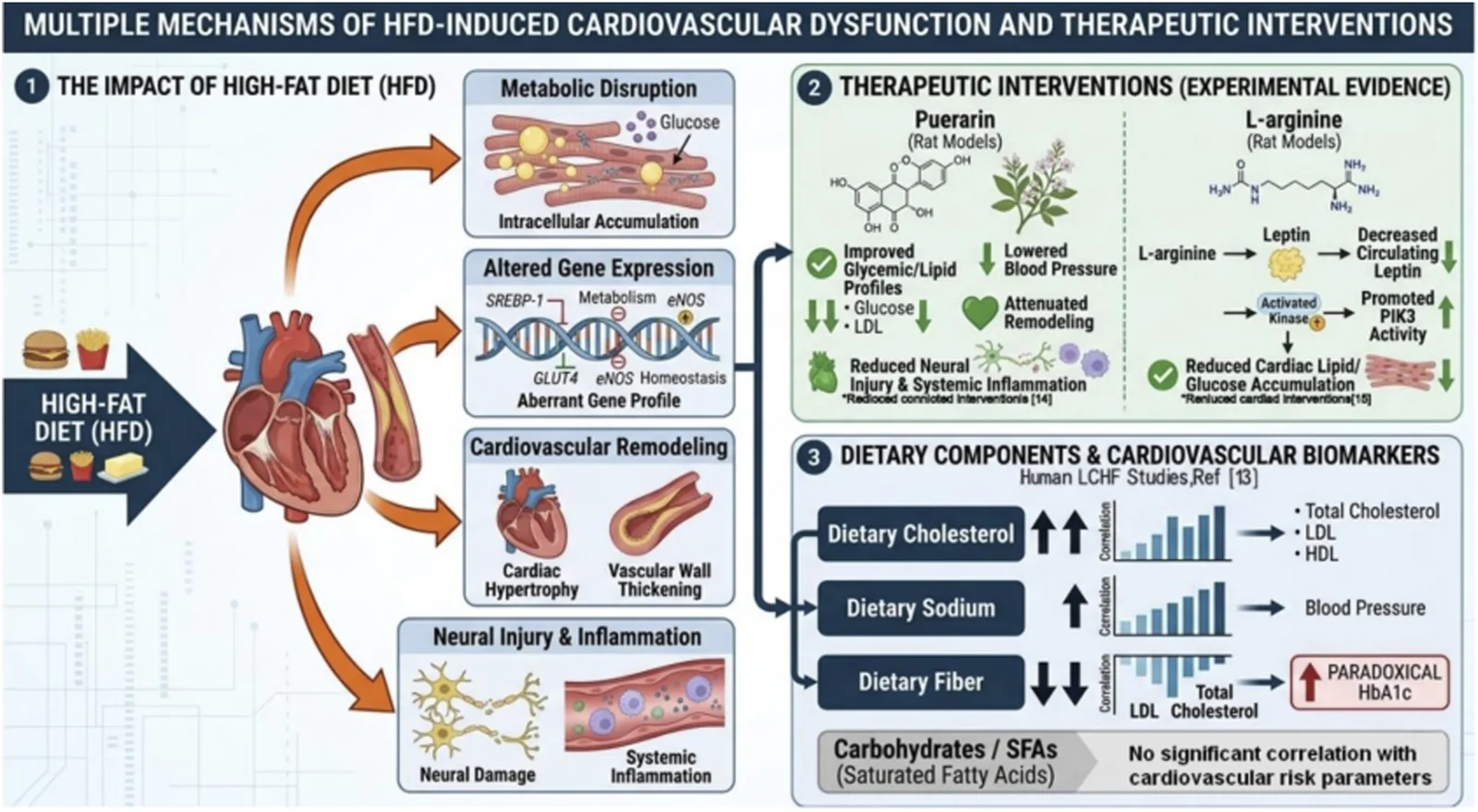
Effects of a high-fat diet on the cardiovascular system through metabolic disturbances and gene expression alterations. A high-fat diet also significantly disrupts cardiac metabolism and alters gene expression. In a metabolic syndrome rat model, administration of puerarin significantly ameliorated systemic inflammatory responses. Supplementation with L-arginine in HFD-fed male Wistar rats reduced circulating leptin levels and enhanced phosphatidylinositol 3-kinase (PIK3) activity.

Clinical and experimental evidence have consistently demonstrated the impact of HFD on coronary health. Observational studies in human populations adhering to a low-carbohydrate, high-fat (LCHF) dietary pattern have revealed distinct associations between specific dietary components and cardiovascular biomarkers. In these populations, dietary cholesterol intake was positively correlated with higher total cholesterol, low-density lipoprotein (LDL), and high-density lipoprotein (HDL) levels. Higher dietary sodium intake was associated with higher blood pressure, while greater dietary fiber consumption was associated with lower LDL and total cholesterol, but paradoxically higher glycated hemoglobin (HbA1c). Notably, no significant associations were observed between carbohydrate or saturated fatty acid (SFA) intake and conventional cardiovascular risk parameters, suggesting a complex interplay between macronutrient composition, lipid metabolism, and glycemic control [13, 16] (Figure 3; Table 1).

#### Integrated mechanisms of high-fat diet-induced cardiovascular injury

The deleterious effects of HFD on the cardiovascular system arise from the convergence of hemodynamic disturbances and metabolic dysregulation. Hemodynamically, HFD impairs cardiac stress responsiveness, disrupts electrophysiological stability, and compromises myocardial contractility, with coronary microvascular injury emerging as an early pathological event preceding overt cardiac dysfunction. Concurrently, HFD induces profound metabolic alterations—including aberrant lipid and glucose accumulation, neurohormonal activation, and inflammatory responses—coupled with the dysregulated expression of genes governing cardiac metabolism and vascular homeostasis. Together, these interrelated mechanisms establish a pro-inflammatory, metabolically unfavorable environment that accelerates cardiovascular remodeling and functional decline, highlighting the multifactorial nature of HFD-induced cardiovascular pathology (Table 1).

#### Epigenetic and molecular mechanisms underlying HFD-Induced cardiovascular injury

Beyond hemodynamic and metabolic disturbances, emerging evidence has highlighted the critical role of epigenetic reprogramming in mediating the long-term effects of HFD. Maternal HFD exposure can alter the fetal epigenome through changes in DNA methylation, histone modifications, and the expression of non-coding RNAs. For instance, HFD-induced hypermethylation of the promoter regions of genes involved in endothelial nitric oxide synthase (eNOS) or antioxidant defense can lead to sustained endothelial dysfunction in offspring. Concurrently, increased oxidative stress and mitochondrial dysfunction in the fetal heart, driven by excessive lipid peroxidation and impaired mitochondrial biogenesis (e.g., decreased PGC-1α expression), can contribute to the impairment of cardiac energetics and contractile function. Furthermore, the placenta acts as a critical mediator when maternal HFD triggers local inflammation and alters nutrient transporter expression, thereby indirectly programming the developing fetal cardiovascular system. These interwoven molecular pathways provide a mechanistic framework for understanding how early-life nutritional insults translate into increased long-term CAD risk.

### Long-term follow-up studies on high-fat diet and coronary health

Despite growing evidence linking HFD consumption to CAD, the long-term effects and underlying pathophysiological mechanisms remain insufficiently elucidated. Current findings are derived from short-term and medium-term studies that primarily established associative rather than causal relationships between HFD and CAD [17, 18]. To advance understanding, well-designed longitudinal studies are required to comprehensively assess the chronic effects of HFD on coronary function and disease progression. Future research should focus on large-scale prospective cohort studies that systematically track dietary patterns, lifestyle factors, and coronary health indicators over time [19]. The regular monitoring of key clinical biomarkers, such as blood lipid levels, inflammatory mediators, oxidative stress parameters, and endothelial function, will enable the more comprehensive delineation of the long-term effects of HFD on coronary physiology and disease progression [20].

Methodologically, prospective cohort studies should include participants across different age groups, sexes, and genetic backgrounds, exposed to varying levels of HFD under well-controlled conditions. Long-term follow-up of clinically relevant endpoints, including CAD incidence, myocardial infarction, and other adverse cardiovascular events, will strengthen causal inference [21, 22]. Furthermore, integrating multi-omics approaches—genomic, transcriptomic, metabolomic, and lipidomic—may further provide insights into potential molecular mechanisms underlying HFD effects on coronary health, thereby providing a basis for developing more precise prevention and treatment strategies [23]. Such multidisciplinary, long-term follow-up studies would not only delineate the long-term pathophysiological effects of HFD but also support the development of personalized dietary recommendations and targeted preventive strategies for CVD management.

## The value of maternal dietary intervention in preventing coronary artery disease in offspring

**FIGURE 4:**
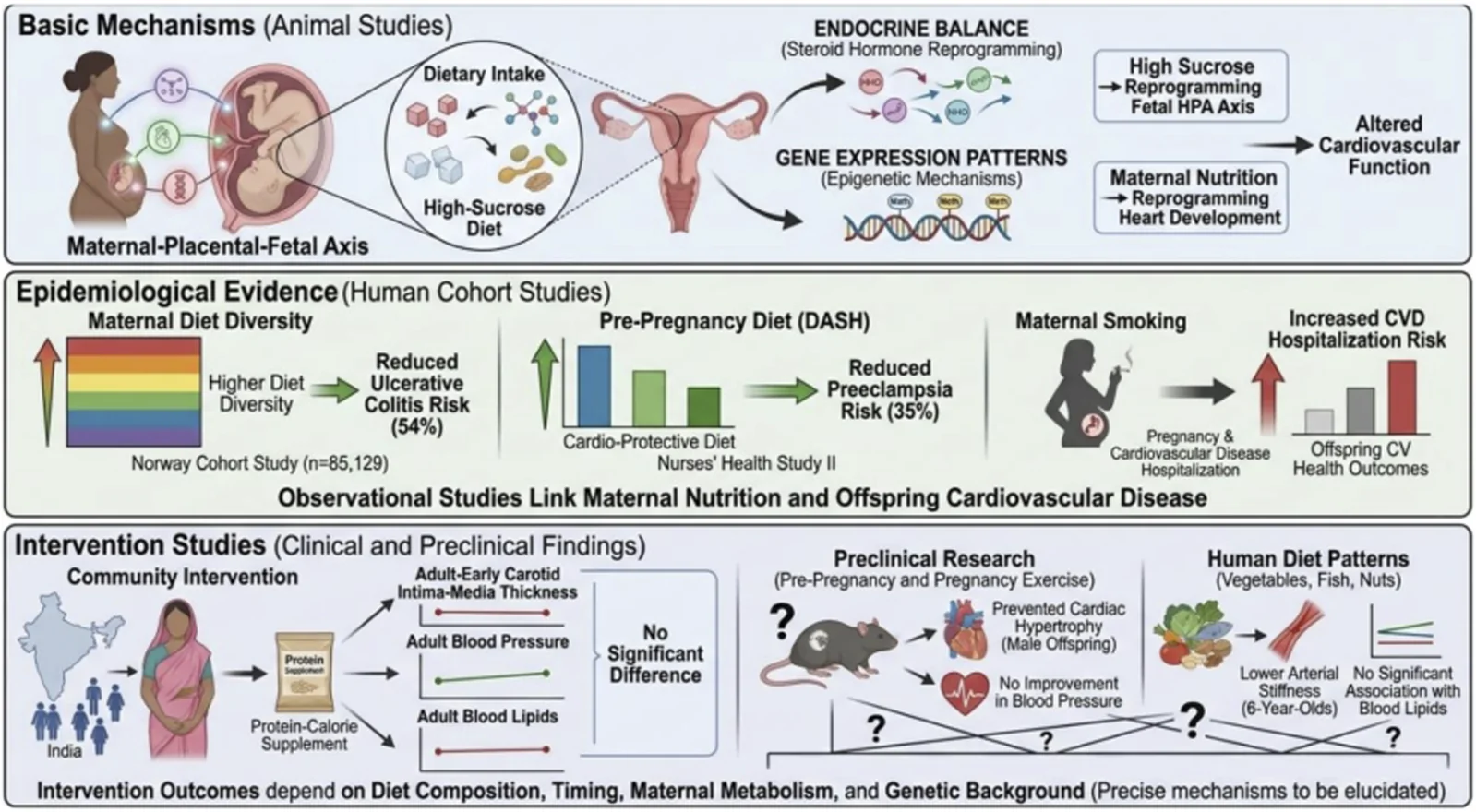
Role of the early-life nutritional environment in shaping the long-term health trajectory of offspring. Maternal dietary patterns during pregnancy not only directly influence fetal intrauterine development, but they also exert profound and lasting programming effects on offspring cardiovascular health through complex biological mechanisms.

The early-life nutritional environment plays a decisive role in shaping the long-term health trajectory of offspring, a concept that has been extensively validated by numerous studies. Maternal dietary patterns during pregnancy not only directly influence fetal intrauterine development, but they also exert profound and lasting programming effects on cardiovascular health through complex biological mechanisms. This chapter systematically elucidates this relationship across three progressive dimensions: the fundamental mechanism level, the epidemiological evidence level, and the intervention research level (Figure 4).

### Long-term effects of maternal pre-pregnancy diet on fetal cardiovascular development

Maternal dietary patterns before and during pregnancy exert profound and long-term effects on fetal development and postnatal cardiovascular health (ranging from 11 to 40 weeks of gestation). Several studies have demonstrated that maternal nutritional status can significantly impact fetal cardiovascular system development by modulating hormonal balance across the maternal–placental–fetal axis and alterations of gene expression patterns. In a rat model, consumption of a high-sucrose diet (HSD) during pregnancy resulted in significant changes in steroid hormone levels in the mother, placenta, and fetus. At embryonic day 19.5, HSD intake increased maternal serum glucocorticoids and aldosterone levels while decreasing placental androstenedione and testosterone concentrations. Concurrently, aldosterone levels were elevated in fetal blood, specific brain regions, and amniotic fluid [24]. These findings indicate that maternal dietary composition can reprogram the fetal hypothalamic–pituitary–adrenal axis and influence cardiovascular regulation mechanisms, potentially predisposing offspring to later-life cardiovascular dysfunction.

Beyond direct cardiovascular effects, maternal diet during pregnancy plays a crucial role in shaping offspring neurodevelopment and behavior. Excessive fructose intake during pregnancy has been shown to impair fetal brain development, leading to neurocognitive deficits. Conversely, maternal voluntary physical exercise (VPE) before and during pregnancy mitigates these adverse effects by attenuating hippocampal oxidative and genotoxic damage, ultimately enhancing memory and learning capacity in offspring [25]. Furthermore, both nutritional deficiency and overnutrition have been associated with increased risk of cardiovascular and metabolic disorders in adult offspring. Severe maternal undernutrition during pregnancy is associated with an increased risk of cardiovascular, renal, and other chronic diseases in adult offspring, while maternal HFD exposure predisposes offspring to neurodevelopmental disorders, such as autism spectrum disorder, attention-deficit hyperactivity disorder, and behavioral impairments [4, 5]. These findings indicate that an appropriate maternal dietary regulation before and during pregnancy is essential for healthy fetal cardiovascular and neurological development and lifelong health status [26–28]. Nutritional imbalances—whether due to deficiency or excess—can disrupt metabolic, hormonal, and epigenetic homeostasis, predisposing offspring to chronic diseases later in life. A synthesis of current findings is presented in Table 2.

**TABLE 2:** Long-term effects of maternal diet and exercise before pregnancy on fetal development.

Study model/type	Core findings	Key indicators/mechanisms	Implications	Citation
Animal study (rats)	Maternal HSD altered steroid hormone levels in the mother, placenta, and fetus	Increased maternal serum glucocorticoids and aldosterone; decreased placental androstenedione and testosterone; elevated fetal blood and brain aldosterone levels	Maternal nutrition can interfere with fetal cardiovascular system programming by modulating the intrauterine hormonal environment	[24]
Animal study (mice)	Maternal exercise before and during pregnancy mitigated the adverse effects of prenatal high-fructose exposure on offspring neurocognitive function	Improved offspring hippocampal biochemical and genotoxic changes, reduced memory impairment	Healthy maternal lifestyles (such as exercise) may counteract diet-induced metabolic and neurodevelopmental abnormalities in offspring	[25]
Human study (systematic review)	Severe maternal undernutrition or HFD during pregnancy increased offspring susceptibility to cardiovascular, renal, and neurodevelopmental disorders, including autism spectrum disorder (ASD)	Mediated by metabolic reprogramming, epigenetic changes, and endocrine dysregulation	Maternal nutritional status before and during pregnancy exerts lifelong health effects on offspring, underscoring the importance of early nutritional intervention	[4, 5]

### Maternal dietary habits during pregnancy and offspring CVD risk

Maternal dietary habits and lifestyle choices during pregnancy exert significant and long-term impacts on offspring cardiovascular health. Several population-based epidemiological studies have explored this relationship from different perspectives. In a Norwegian nationwide cohort study involving 85,129 children (the children were born between 1999 and 2009, with follow-up through December 31, 2021), the average follow-up time was 16.1 years, and the average age of diagnosis was 12.4 years. Greater maternal dietary diversity during pregnancy was associated with a significantly reduced risk of ulcerative colitis (UC) in offspring, suggesting that diverse maternal nutrition may beneficially modulate offspring immune and inflammatory responses in the fetus. Specifically, children born to mothers in the high-diversity group exhibited a 54% reduction in UC risk compared with those in the low-diversity group (adjusted HR 0.46, 95% CI 0.25–0.87) [29].

Maternal adherence to cardioprotective dietary patterns before and during pregnancy has been linked to reduced risks of gestational and cardiovascular complications. In a cohort of 16,892 women with singleton pregnancies from the Nurses' Health Study II, higher pre-pregnancy adherence to the American Heart Association (AHA) dietary recommendations and the Dietary Approaches to Stop Hypertension (DASH) diet was associated with significantly lower risk of preeclampsia. Women in the highest quintile of DASH adherence exhibited a 35% reduction in preeclampsia risk compared with those in the lowest quintile (RR 0.65, 95% CI 0.48–0.87) [30]. Conversely, adverse maternal behaviors, such as smoking during pregnancy, have been significantly associated with increased CVD risk in offspring. A population-based cohort study found that maternal smoking during pregnancy was associated with a significantly higher rate of cardiovascular-related hospitalization in offspring (1.3% vs. 0.6%, OR 2.1, 95% CI 1.5–2.9; *P* < 0.001) [31]. The observed increase in cardiovascular morbidity may be attributed to *in utero* exposure to nicotine and oxidative stress, leading to endothelial dysfunction, vascular remodeling, and adverse metabolic programming. Collectively, these studies provide robust epidemiological evidence that maternal dietary patterns, nutrient diversity, and lifestyle behaviors (such as smoking) during pregnancy profoundly impact offspring cardiovascular outcomes [32]. Therefore, ensuring optimal maternal nutrition, characterized by balanced macronutrient intake, reduced processed fat consumption, and avoidance of harmful substance exposures, during pregnancy may represent a key strategy in preventing CVD risk in offspring. A synthesis of these findings is presented in Table 3.

**TABLE 3:** Maternal dietary habits during pregnancy and offspring cardiovascular disease risk.

Study population/type	Core findings	Risk ratio/association strength	Conclusion	Citation
Norwegian nationwide cohort	Greater maternal dietary diversity during pregnancy was associated with a reduced risk of UC in offspring	High vs. low diversity: HR = 0.46 (0.25–0.87)	Prenatal dietary diversity may enhance immune development in offspring, thereby contributing to improved long-term cardiovascular and metabolic health	[29]
Nurses' health study II	High pre-pregnancy AHA/DASH adherence was linked to lower preeclampsia risk	Highest vs. lowest DASH quintile: RR = 0.65 (0.48–0.87)	Pre-pregnancy heart-healthy diets may prevent hypertensive disorders of pregnancy and confer cardiovascular benefits to both mother and child	[30]
Population-based cohort	Maternal smoking during pregnancy was significantly associated with an increased rate of cardiovascular-related hospitalization in offspring	Smokers’ offspring (1.3%) vs. non-smokers’ offspring (0.6%): OR = 2.1 (1.5–2.9)	Prenatal smoking is a significant risk factor for adverse cardiovascular outcomes in offspring and should be strongly discouraged	[31]

### Impact of dietary intervention during pregnancy on offspring coronary health

The impact of maternal dietary intervention during pregnancy on the cardiovascular health of offspring has emerged as a key focus in current perinatal and cardiovascular research. Studies investigating this relationship have explored both direct nutritional interventions during pregnancy and long-term cardiovascular outcomes in offspring. In a community-based, non-randomized controlled intervention trial conducted in India, protein-calorie food supplementation was provided to pregnant and lactating women, as well as to their children under 6 years of age, across 15 intervention villages without malnutrition conditions. Long-term follow-up at a mean offspring age of 21.6 years revealed no significant differences in key cardiovascular risk factors—including carotid intima-media thickness (cIMT), arterial stiffness, systolic blood pressure, body mass index (BMI), LDL cholesterol, or fasting insulin levels compared with controls [33].

In preclinical studies, exercise and dietary interventions have demonstrated more specific effects on offspring cardiovascular outcomes. In diet-induced obese female C57BL/6 mice, treadmill exercise initiated 1 week before and during pregnancy prevented pathological cardiac hypertrophy and systolic dysfunction in 8-week-old male offspring. However, the intervention did not improve hypertension, indicating that the mechanisms underlying blood pressure regulation may differ from those regulating cardiac function and structure [34]. Furthermore, studies have shown that maternal adherence to specific dietary patterns, particularly those rich in vegetables, fish and oils, nuts, soy, and high-fiber grains, during pregnancy was associated with reduced pulse wave velocity—a marker of arterial stiffness—in offspring at 6 years of age. However, no significant associations were observed with blood lipid or insulin levels [35]. These findings suggest that the effects of maternal dietary intervention during pregnancy on offspring coronary health are complex and context-dependent. Variation in dietary composition, intervention timing and duration, maternal metabolic state, and genetic background may contribute to different cardiovascular outcomes in offspring, necessitating further longitudinal and mechanistic research to elucidate the precise pathways through which maternal nutrition programs cardiovascular development and disease susceptibility in offspring. A summary of relevant findings is presented in Table 4.

**TABLE 4:** Impact of maternal dietary and lifestyle intervention during pregnancy on offspring coronary health.

Study type/subject	Intervention	Impact on offspring cardiovascular health	Conclusions and implications	Citation
Human community-based trial (India)	Protein-calorie supplement for pregnant and lactating women	No significant effect on cIMT, arterial stiffness, BP, BMI, LDL, or fasting insulin in offspring at ∼21.6 years of age	Simple calorie-protein supplementation during pregnancy may have limited efficacy in improving long-term cardiovascular outcomes in offspring, underscoring the need for more comprehensive nutritional interventions	[33]
Human cohort study (generation R)	Assessment of maternal prenatal dietary patterns	Maternal adherence to a vegetables, fish, and oils dietary pattern was associated with lower pulse wave velocity in offspring at age 6, whereas no significant effect was observed on lipids or insulin levels	Specific healthy maternal dietary patterns during pregnancy may confer early vascular protective effects in offspring; however, these benefits appear to be pattern- and nutrient-specific	[35]
Animal study (mice)	Treadmill exercise intervention in diet-induced obese dams before and during pregnancy	Prevented pathological cardiac hypertrophy and dysfunction in male offspring at 8 weeks of age, but did not improve hypertension	Maternal exercise exerts protective effects on offspring cardiac structure and function, but BP regulation may involve different mechanisms	[34]

## Evidence discrepancies and perspectives on the impact of high-fat and pregnancy diets on coronary artery disease

### Conflicting evidence on the effects of high-fat diet and coronary health

The impact of HFD on coronary health remains a subject of ongoing debate, with studies reporting conflicting findings. Some studies suggest that HFD intake, particularly SFAs, increases circulating LDL cholesterol concentration, thereby increasing the risk of atherosclerotic CVD (ASCVD). However, other studies have provided different interpretations of these associations. A study proposed the “Homeoviscous Adaptation to Dietary Lipids (HADL) model,” which suggests that SFA-induced LDL cholesterol elevation may reflect a physiological, adaptive homeostatic response aimed at maintaining cell membrane fluidity and cellular function, rather than a pathological response in metabolically healthy individuals. However, this adaptive mechanism may become dysregulated in individuals with metabolic disorders such as insulin resistance and other ASCVD risk factors, potentially transforming a physiological adaptation into a pathophysiological process [36].

**FIGURE 5:**
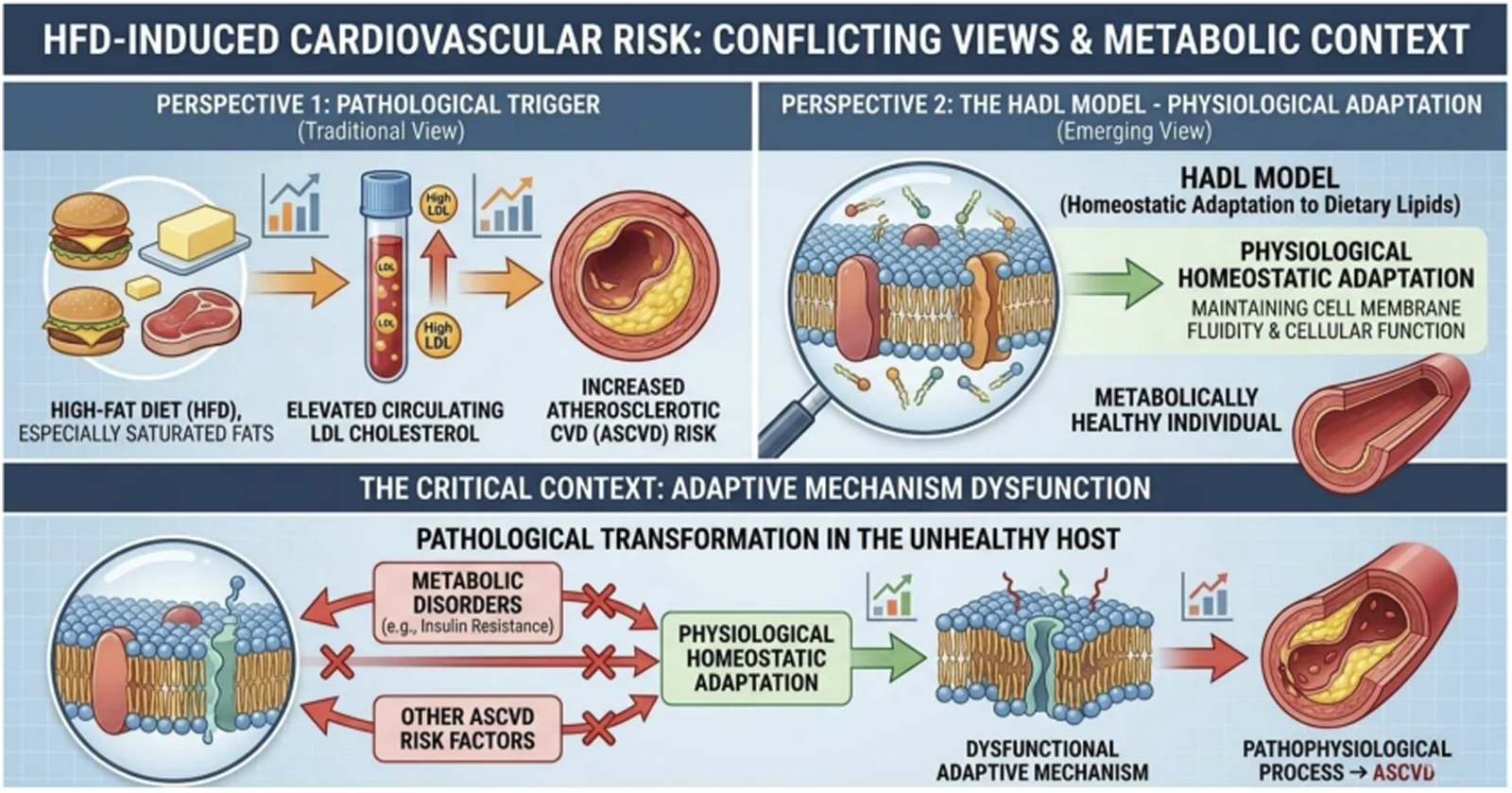
The impact of a high-fat diet on coronary artery health remains controversial. High fat diet intake elevates circulating low-density lipoprotein cholesterol levels, thereby increasing the risk of atherosclerotic arterial cardiovascular disease. However, other studies propose alternative explanations, suggesting that the saturated fatty acid-induced elevation of low-density lipoprotein cholesterol may reflect a physiological homeostatic adaptation response aimed at maintaining cell membrane fluidity and cellular function, rather than a pathological reaction in metabolically healthy individuals.

Further controversy centers on the impact of different types of fatty acids on CVD risk. Although several studies have reported that substituting SFA with unsaturated fats—particularly polyunsaturated fatty acids (PUFAs)—reduces CVD risk, others have demonstrated significant interindividual variation in serum LDL-cholesterol response to dietary fatty acids. This variability may be attributed to genetic, metabolic, or gut microbiome-related differences among individuals, making it difficult to establish unified dietary recommendations for CVD prevention [37]. Collectively, these findings underscore the complexity of the relationship between HFD and coronary health, indicating that a one-size-fits-all dietary approach may be inadequate. Further mechanistic studies are warranted to delineate the interplay between dietary lipids, metabolism, and genetic background, thereby facilitating individualized dietary interventions for optimizing cardiovascular outcomes (Figure 5).

### Different perspectives on maternal diet and offspring coronary health

The impact of maternal diet during pregnancy on offspring coronary health also remains controversial. Several studies suggest that maternal dietary patterns and nutrient composition during pregnancy exert significant effects on offspring cardiovascular health. For instance, high maternal carbohydrate intake during pregnancy has been associated with increased blood pressure in offspring, while higher protein intake has been negatively correlated with offspring cIMT, suggesting a protective effect on vascular integrity [38]. Similarly, maternal adherence to a Mediterranean diet rich in fruits, vegetables, whole grains, fish, and unsaturated fats during pregnancy has been linked to lower BMI z-score, waist circumference, and blood pressure in offspring during childhood, suggesting a protective effect against early cardiovascular risk development [39–41].

In contrast, other studies have reported minimal or no significant association between maternal diet and offspring cardiovascular health. Evidence from the Generation R cohort study involving 2,592 mother-child pairs revealed that most maternal dietary patterns during pregnancy were not significantly associated with key cardiometabolic health indicators—including pulse wave velocity, blood pressure, insulin, HDL-cholesterol, and triglycerides—in offspring at 6 years of age, after adjusting for potential confounders such as sociodemographic and lifestyle factors. Only the “vegetables, fish, and oils” dietary pattern was associated with lower pulse wave velocity, suggesting a potential but limited vascular benefit [35]. These discrepancies across studies may be attributed to variations in study populations, dietary assessment methods, genetic and environmental factors, and statistical adjustment for confounders, thereby precluding the establishment of definitive conclusions regarding the causal relationship between maternal diet and offspring coronary health. At the same time, when analyzing conclusions, offspring should be stratified based on their age or developmental stage. Therefore, further large-scale, longitudinal studies are warranted to elucidate the precise impact of maternal nutrition during pregnancy on offspring coronary health and long-term cardiovascular outcomes [42–44]. A summary of current evidence is presented in Table 5.

**TABLE 5:** Differing perspectives on the impact of maternal diet during pregnancy on offspring coronary health.

Viewpoint tendency	Representative research/finding	Key evidence and interpretation	Citation
Supports significant impact	High maternal prenatal carbohydrate intake was associated with increased offspring BP, whereas protein intake was linked to decreased offspring cIMT	Suggests an association between specific nutrient intake and early cardiovascular risk markers in offspring	[38]
Supports significant impact	Maternal mediterranean diet during pregnancy was associated with lower childhood BMI, waist circumference, and BP in offspring	Suggests a healthy overall dietary pattern may exert long-term protective effects on offspring cardiovascular health	[39–41]
Suggests limited or no significant association	Generation R cohort: Most maternal prenatal dietary patterns showed no significant association with several cardiometabolic indicators in offspring at age 6 after statistical adjustments	Only the “vegetables, fish, and oils” dietary pattern was associated with lower arterial stiffness (reduced pulse wave velocity)	[35]

### Sex-specific programming effects of maternal diet

A critical consideration in the developmental programming of CAD is the sex-specific susceptibility of offspring. Experimental evidence consistently shows that male and female offspring exhibit distinct cardiovascular and metabolic phenotypes in response to maternal HFD. For example, Alsiraj et al. found that male offspring from HFD-fed dams showed blunted stress responses, while female offspring exhibited reduced baseline cardiovascular function [2]. These differences may stem from sex-specific alterations in placental function, hormone signaling (e.g., estrogen’s protective role), and epigenetic modifications. The current understanding of this area remains nascent, and future studies must be adequately powered to analyze outcomes by sex to make possible the development of targeted and personalized preventive strategies.

## Discussion

The relationship between maternal diet during pregnancy and offspring cardiovascular health represents a critical research area with significant implications for public health and disease prevention. Although several studies have highlighted potential associations between maternal nutrition and offspring cardiometabolic outcomes, significant methodological limitations persist, including limited sample sizes and population diversity, as well as inadequate control of confounding variables. Future research should prioritize large-scale, multiethnic, and socioeconomically diverse cohorts to enhance the external validity and generalizability of findings [45–48]. Beyond conventional assessments of nutrient intake, future studies should investigate the effects of dietary patterns, meal timing, and overall dietary quality throughout pregnancy on offspring cardiovascular outcomes [41, 49, 50]. For example, exploring maternal nighttime eating behavior during pregnancy may reveal novel mechanisms underlying fetal metabolic programming. Integrating advanced perinatal imaging and monitoring technologies, such as fetal echocardiography, Doppler ultrasound, and noninvasive fetal cardiac MRI, may also provide critical mechanistic insights into the early physiological effects of maternal diet on fetal cardiovascular development. Long-term follow-up of offspring from birth through adolescence and adulthood is essential to elucidate the impact of prenatal dietary exposures on long-term CVD risk [51]. Additionally, interventional studies providing structured nutritional guidance and maternal dietary modifications during pregnancy are needed to establish causal relationships and the efficacy of dietary interventions in improving offspring cardiovascular health, thereby providing more reliable evidence for developing dietary guidelines aimed at reducing the risk of CVDs in offspring [52–54].

Furthermore, embracing emerging technologies will be crucial for deciphering the complex biology of developmental programming. The application of single-cell and spatial transcriptomics can be used to explore the cellular heterogeneity within the developing heart and vasculature, revealing cell-type-specific responses to maternal diet. Integrating multi-omics data (genomics, epigenomics, transcriptomics, metabolomics) through systems biology approaches holds the potential to make it possible to build predictive models of offspring CVD risk. Additionally, the use of placental organoids and advanced imaging techniques offers new avenues for exploring placental function non-invasively. Ultimately, these approaches will pave the way for precision nutrition, enabling the design of personalized dietary interventions based on maternal genotype, metabolic profile, and microbiome composition.

## Limitations

This review synthesized evidence primarily derived from animal experiments and observational studies, which may limit its direct applicability to human populations. Many existing studies are limited by potential confounding factors (such as genetic background and lifestyle behaviors) and lack of long-term follow-up data. Furthermore, inter-individual variability in dietary responses may limit the generalizability of findings. Therefore, well-designed prospective cohort studies and randomized controlled trials are warranted to validate causal relationships and to elucidate the precise underlying mechanisms linking maternal nutrition to offspring cardiovascular health.

## Conclusion

Preclinical studies in animal models strongly suggest that maternal HFD exposure pre-pregnancy can predispose offspring to CAD in adulthood through mechanisms involving epigenetic reprogramming, metabolic dysregulation, and endothelial dysfunction. While observational studies in humans support these associations, evidence for direct causality remains limited. Therefore, the translational significance of these mechanistic findings requires validation through carefully designed prospective cohort studies and, where feasible, randomized controlled trials. Targeted nutritional interventions during pregnancy, particularly when initiated before conception or during early gestation, have the potential to counteract these adverse programming effects. However, the long-term efficacy and underlying mechanisms of such interventions remain incompletely understood. Future large-scale, prospective cohort studies integrating multi-omics approaches are warranted to clarify the causal links between maternal nutritional exposures and offspring cardiovascular outcomes.
